# Acupotomy treatment for finger joint contracture after immobilization: Case report

**DOI:** 10.1097/MD.0000000000024988

**Published:** 2021-03-12

**Authors:** Sang-Hoon Yoon, Jiyun Cha, Eunji Lee, Byeongjo Kwon, Kyongha Cho, Sungha Kim

**Affiliations:** a Chung-Yeon Korean Medicine Clinic, 404, Nonhyeon-ro, Gangnam-gu, Seoul; b Korea Institute of Oriental Medicine, 1672 Yuseong-daero, Yuseong-gu, Daejeon; c Chung-Yeon Korean Medicine Hospital, 64 Sangmujungang-ro, Seo-gu, Gwangju; d Baros Korean Medicine Clinic, 4, Muwang-ro 16-gil, Iksan-si, Jeollabuk-do, Korea.

**Keywords:** acupotomy, case report, immobilization, joint contracture

## Abstract

**Introduction:**

Contractures frequently occur in the finger joints after immobilization. This report describes the effect of acupotomy treatment in patients with joint contracture due to immobilization of the finger joints.

**Patient concerns and clinical findings:**

Case 1 was of a 39-year-old male patient who had flexion limitation of the left thumb and difficulty in grasping. Case 2 was of a 41-year-old female patient who had flexion limitation of the right index finger and difficulty in typing. Stiffness occurred after tendon repair surgery and cast immobilization in both cases. In Case 1, the patient had limited flexion movement of the first metacarpophalangeal and interphalangeal joints after 5 weeks of immobilization of the left thumb in a cast. In Case 2, the patient had limited flexion movement after 3 weeks of immobilization of the second proximal interphalangeal joint of the left hand in a cast.

**Diagnosis, interventions, and outcomes:**

We diagnosed both patients with finger joint contracture due to immobilization. Conservative treatment for approximately 4 weeks did not lead to improvement in either patient. Acupotomy is the key treatment for improving movement in Korean Medicine. Therefore, acupotomy was performed, and joint stiffness markedly improved without adverse events. Both patients reported that the daily use of the damaged fingers became comfortable.

**Conclusion:**

We found that acupotomy may be effective for finger joint contracture due to improper immobilization. We suggest it as a simple and safe treatment for joint contracture.

## 1 Introduction

When an injury occurs, fixation using casts and splints is a good treatment option to help recovery.^[1]^ However, it is known that excessively long or incorrect immobilization might lead to side effects such as joint contracture, muscle atrophy, or ligament weakening as an adaptive response.^[1–3]^ For ankle joints immobilization, the prevalence of plantarflexion contracture has been reported at 77% immediately after cast removal and at 22% 2 years after removal.^[4,5]^ Importantly, joint stiffness caused by contracture may lead to severe discomfort in daily life.^[6]^ Contractures frequently occur in the finger joints.^[2]^

It is generally known that approximately 3 to 6 months of appropriate rehabilitation are needed for stiffed fingers to recover their normal movement range.^[7]^ Although stretching is widely used as a conservative treatment for contractures, evidence for its effectiveness has not been established.^[8]^ Surgery is the final treatment option for contractures, but patients with contracture after injury or surgery may be reluctant to undergo reoperation.^[9,10]^

Acupotomy can be an alternative treatment. Developed from traditional acupuncture in oriental medicine, it is a type of invasive needling treatment but uses a thick, flat-head, safer needle.^[11]^ The use of acupotomy has been reported for the treatment of various musculoskeletal disorders including adhesive arthritis of the shoulder by releasing tissue adhesion and promoting blood circulation and recovery around the joints.^[12,13]^

Here, we report the cases of 2 patients with immobilization-caused contractures who were easily and safely treated with acupotomy. We consider sharing this experience important, as the patients showed finger motion recovery and treatment satisfaction and, to our knowledge, similar findings have not been previously reported. This case report was compiled in accordance with the CAse REport Guidelines.^[14]^

## 2 Case presentation

The Institutional Review Board of Chung-Yeon Korean Medicine Hospital, Gwangju, provided ethical approval and exemption from review for this study (CYIRB 2019-10-002) because the risk to the subjects was minimal, and the study did not include subjects in a vulnerable environment or collected sensitive personal information of the subjects.

Consent for research and publication was obtained from both patients. Because of the physical distance constraints, we secured verbal consent over the telephone instead of written consent. The patient of the first case agreed to the publication of his photographs.

### 2.1 Case 1

A 39-year-old man working as an office employee was injured in his left hand in a car accident on October 25, 2017. He was diagnosed with lacerations of the tendon of the extensor pollicis brevis and the dorsum of the metacarpal of the left thumb and underwent tendon repair surgery on the day of accident. For Korean medicine postoperative care, he visited our hospital on October 30, wearing a cast.

**Figure 1:**
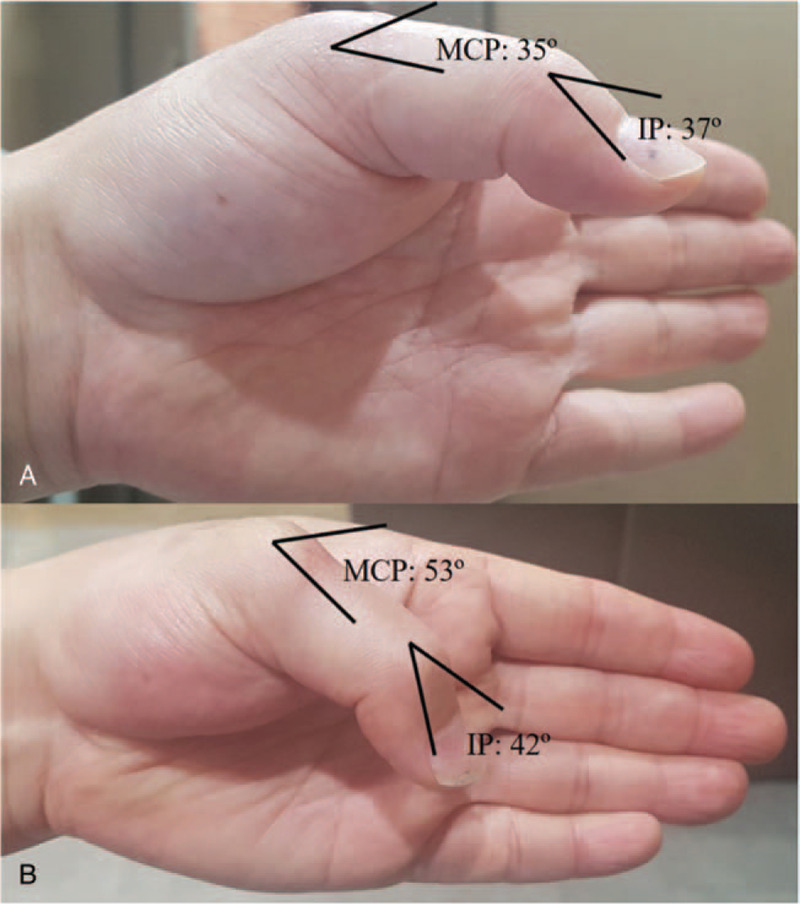
(A): Before the first acupotomy treatment (B): After the fourth acupotomy treatment. IP = interphalangeal, MCP = metacarpophalangeal.

On November 29, after 5 weeks of immobilization, the cast was removed. However, contracture and flexion limitation occurred on the first left metacarpophalangeal (MCP) and interphalangeal (IP) joints (Fig. 1). Because of flexion limitation, the patient had difficulty in grasping small objects with his left hand. He felt tension and limitation of the extensor tendons of the first MCP and IP joints when attempting to flex his left thumb. Passive flexion also caused motion limitation and pain. The flexion angles of the MCP and IP joints were 35° and 37°, respectively (normal range: 55° for first MCP joint flexion/80° for first IP joint flexion). Similar angles were noted for the passive flexion of both joints.

In addition to flexion limitation, pain and tenderness were observed in the joints near the extensor tendons of the first MCP and IP joints. Acute inflammation or infection was excluded because there was no redness, swelling, or fever. The patient was healthy and was not using any medication.

**Figure 2:**
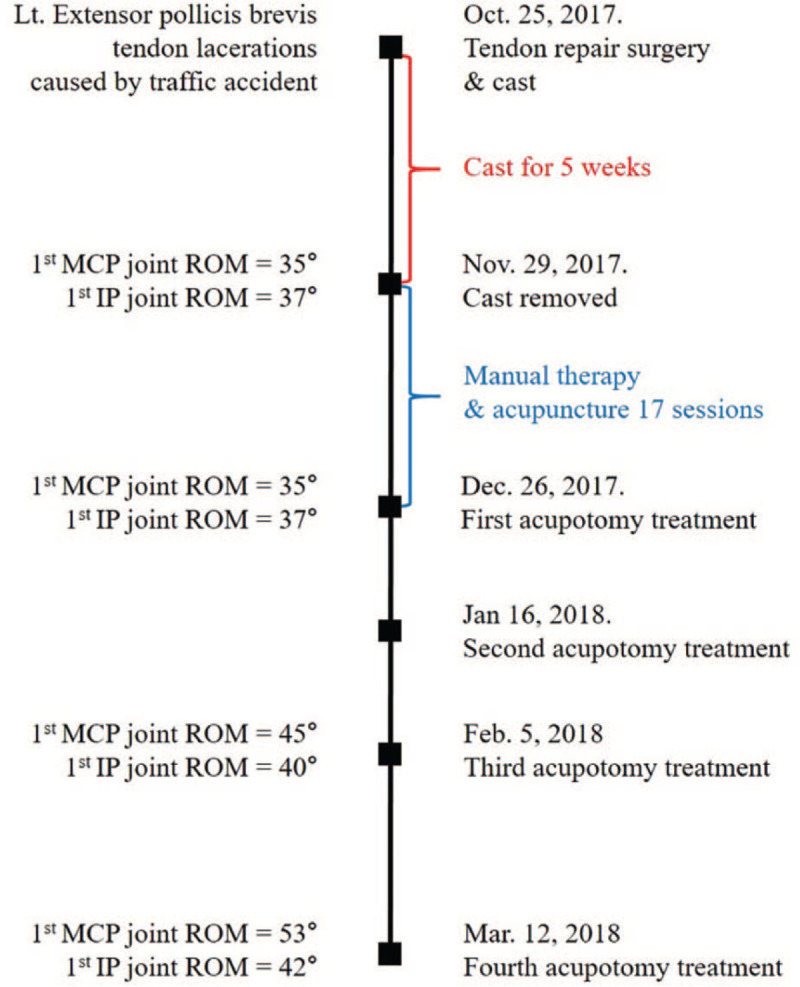
Timeline of Case 1. IP = interphalangeal, MCP = metacarpophalangeal, ROM = range of motion.

We diagnosed his symptom as joint contracture after immobilization. Manual therapy, acupuncture, and self-stretching exercises were performed 17 times for 4 weeks to improve the range of motion (ROM) of the MCP and IP joints of the thumb, but the motion was not improved. Thus, we decided to apply acupotomy treatment as a novel intervention (Fig. 2).

**Figure 3:**
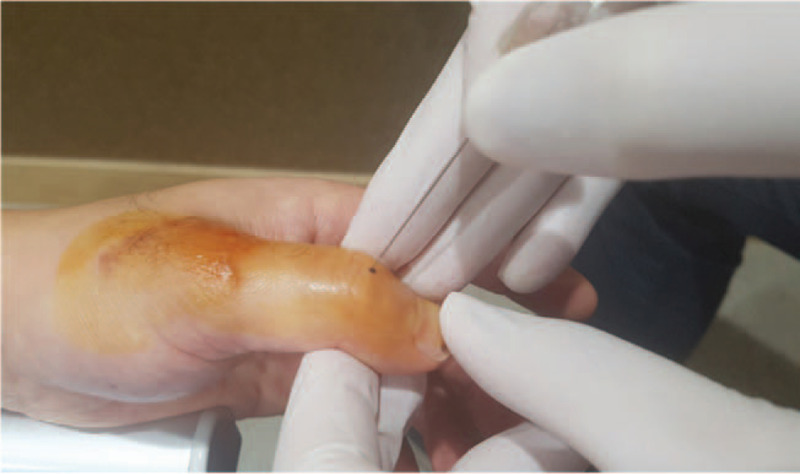
Acupotomy at the joint capsule of the interphalangeal joint.

Acupotomy treatment was performed by a licensed Korean medicine doctor who had 9 years of clinical experience and 7 years of acupotomy treatment experience. Acupotomy (scalpel width: 0.5 mm, length: 50 mm; Dongbang Dochim, Dongbang Medical, Boryeong, Korea) treatment was applied on the articular capsule around the extensor tendon of the first MCP and IP joints. The doctor marked the areas with the greatest pain and tenderness when the patient bent the finger with a surgical marker. These areas were sterilized with a povidone swab stick before they were anesthetized with 0.2 mL Bufonis Venenum phamacoacupuncture.^[15]^ To avoid nerve, blood vessel, and tendon injury, the tip of the blade was placed parallel to the extensor tendon of the finger (Fig. 3). The acupotomy blade was approached until it contacted the articular capsule and was retrieved after 2–3 sessions of lifting–thrusting manipulation to maximize stimulation. The doctor released the adhesions of the articular capsule, taking care not to dissect the extensor tendon. In addition to the acupotomy treatment, manual therapy was applied. The acupotomy treatment procedure corresponded to the Standards for Reporting Interventions in Clinical Trials of Acupuncture criteria (Supplemental Digital Content).^[16]^

The doctor performed acupotomy 4 times, once a month. The patient steadily visited the hospital on the scheduled date and received all treatments. After the second treatment, the flexion angles of the MCP and IP joints improved to 45° and 40°, respectively. Finally, after the fourth treatment (15 weeks after surgery), the flexion angles of the MCP and IP joints improved to 53° and 42°, respectively. The patient stated: “When I took off my watch, I felt very uncomfortable that my finger wasn’t bent. However, at present, the finger movement and pain are much improved, and I feel comfortable.” No adverse events occurred during the treatment period.

### 2.2 Case 2

A 41-year-old woman working as a professor experienced a cut over her right palm from a broken glass on March 5, 2018. This accident damaged the palmar-side flexor tendon and artery of the proximal interphalangeal (PIP) joint of the index finger, and she underwent tendon repair surgery. After 3 weeks of immobilization, the cast was removed, and finger contracture was observed. Joint contracture of the right index finger persisted during a 4-week course of physical and manual therapy. Therefore, the patient visited our hospital on April 26, 2018.

**Figure 4:**
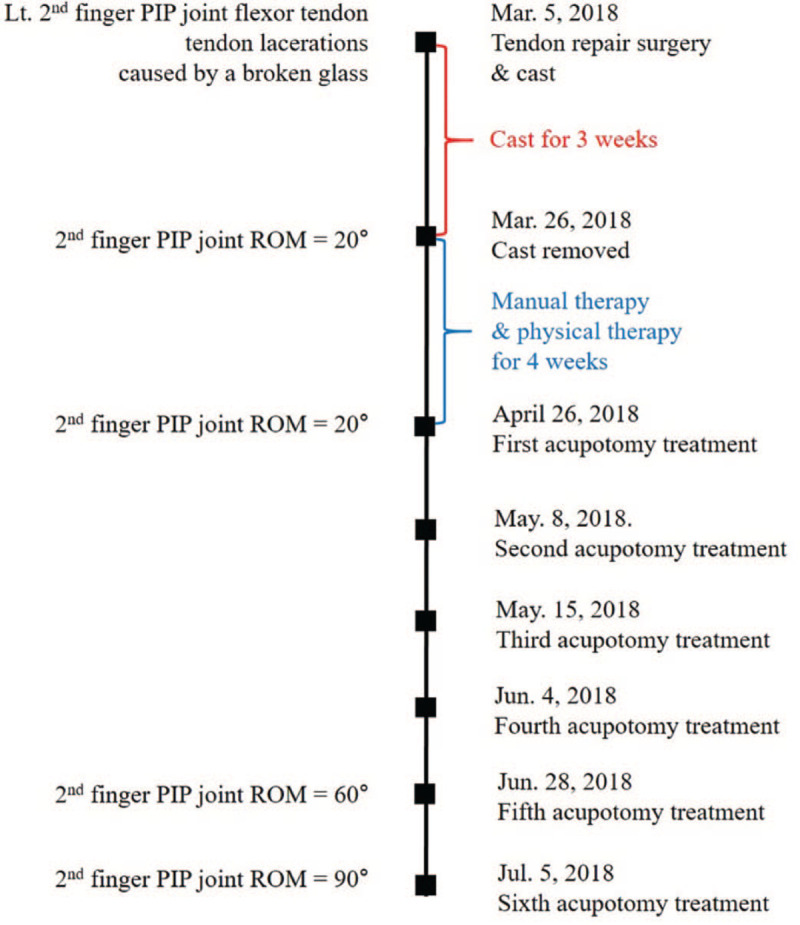
Timeline of Case 2. PIP = proximal interphalangeal, ROM = range of motion.

On examination, the motion of her right second PIP joint was limited, and she was unable to type on a keyboard. The flexion angle of the right second PIP joint was 20°(normal range: 100° for the second PIP joint flexion), and tenderness and swelling of the joint were noted. The patient was healthy and using no medication. We diagnosed her symptom as joint contracture after immobilization (Fig. 4).

Acupotomy treatment was performed 6 times, every 7 to 10 days, from April 26 to July 5. This treatment was conducted by a licensed Korean medicine doctor with 10 years of clinical experience and 7 years of acupotomy treatment experience. Acupotomy (scalpel width: 0.5 mm, length: 50 mm; Dongbang Dochim, Dongbang Medical) treatment was applied on the articular capsule around the flexor tendon sheath of the second PIP joint and the adhered skin. The doctor marked the areas with the greatest pain and tenderness when the patient bent the finger with a surgical maker. These areas were sterilized with a povidone swab stick before anesthesia was commenced with 0.2 mL Bufonis Venenum phamacoacupuncture. To avoid nerve, blood vessel, and tendon injury, the blade tip was placed parallel to the flexor tendon of the finger. The doctor inserted the acupotomy blade into the joint capsule, twisted and pulled it 2 to 3 times, and pulled it out. The doctor released the adhesions of the articular capsule, taking care not to dissect the flexor tendon. Besides acupotomy treatment, acupuncture was applied. The acupotomy treatment procedure corresponded to the Standards for Reporting Interventions in Clinical Trials of Acupuncture criteria.^[16]^

The patient steadily visited the hospital on the scheduled date and received all treatments. After the second acupotomy treatment, the ROM of the finger joint improved, and it was possible for the patient to type on the keyboard. However, numbness of the finger continued. After 2 more treatments, the patient reported that the finger numbness was improving. On June 28, the color of the fingers was found to have markedly recovered, and the flexion angle had improved to 60°.

After the last treatment (17 weeks after surgery), she could flex the PIP joint of the second finger of her right hand by 90°. No side effect occurred during the treatment. Before the last treatment, the patient stated: “I could not swim because of the thickened finger scar after surgery. With acupotomy treatment, the scar has been thinning, and now I can tread water with my fingers. The hospital recommended reoperation, but I am satisfied with the current treatment and decided not to undergo the procedure.” On March 13, 2019, 1 year after the surgery, she visited our hospital for regular observation and reported that she had regained good use of her finger.

## 3 Discussion

We reported these cases to suggest an effective and efficient treatment modality for post-immobilization joint contracture. In both cases, joint contracture occurred after surgery and immobilization (5 weeks and 3 weeks, respectively) due to traumatic tendon damage. We performed acupotomy to these patients and report its effectiveness.

Finger contracture can occur following adhesion and shortening of the articular capsule due to muscle tension, Dupuytren's contracture, skin wounds, degenerative changes such as osteoporosis, intravitreal vitreous, and prolonged immobilization.^[6]^ The articular capsule is most importantly implicated in contractures among the arthrogenic components such as the bone, cartilage, synovial membrane, capsule, and ligaments.^[17,18]^ When the connective tissue is injured, tissues are continuously removed, replaced, and reorganized during healing.^[19]^ Proper immobilization protects the damaged arthrogenic tissue and aids recovery. However, prolonged or improper immobilization rather harms the articular capsule; it causes synovial hypertrophy with fibrosis and fixed tightening of the tissue around the joint. As a result, stiffness and contracture lead to motion limitation in daily life. It is generally known that casts extending beyond the metacarpal heads often cause finger stiffness.^[20–22]^

In general, there are some measures that can prevent contracture including physical exercise prior to immobilization, avoiding prolonged immobilization, early ROM exercises, and proper positioning.^[23,24]^ For contracture and motion limitation, stretching is the most common conservative treatment in the clinic. Infrared or ultrasound heating therapy, passive motion training, neuromuscular electrical stimulation, and botulinum toxin injection may also be applied.^[25,26]^ However, some patients do not respond to conservative treatments and often experience recurrence.^[27,28]^ Besides, in recent systematic reviews, stretching did not show a clinically important effect on joint contracture including on the post-immobilization results.^[8,22,29]^ Another general conservative treatment, botulinum toxin injection, has been reported to cause autonomic side effects such as mouth dryness.^[30]^

Continuous joint contracture is primarily treated with incision of adhered and hypertrophic tissues. A previous study showed increased ROM on extension after incision of the posterior capsule in knee joints with immobilization-induced flexion contractures in a rat model.^[31]^ However, surgery is burdened by the required anesthesia, recovery, and rehabilitation. In addition, patients with postoperative or post-immobilization sequelae are generally reluctant to undergo further surgery. Thus, it is necessary to consider treatments that involve incision but are less invasive for post-traumatic joint contracture.

Acupotomy was developed from traditional acupuncture. A small needle with a blade tip is used to cut adhesions and relieve tissue tension.^[32,33]^ Without anesthesia, acupotomy is inexpensive, effective, and simpler and safer than surgery.^[34,35]^ It is generally used to treat chronic musculoskeletal diseases such as cervical spondylosis, osteoarthritis, and spinal stenosis.^[36]^ Particularly, acupotomy has been reported to relieve joint contracture and stiffness. In a meta-analysis of eight randomized controlled trials on acupotomy for shoulder adhesive capsulitis with stiffness, the acupotomy groups showed significantly greater improvement on the visual analog scale and Constant–Murley score than the control groups.^[37]^ Acupotomy treatment also led to a statistically significant reduction in 5-hydroxytryptamine and prostaglandin E2 in adhesive capsulitis of the shoulder in a rat model.^[38–40]^

Based on the previous studies, we assumed that acupotomy could improve post-immobilization contracture. As a result of acupotomy applications (4 and 6 times, respectively), finger flexion movement and daily finger use, such as during swimming, improved. Moreover, it led to improvement of swelling and pain, which are generally associated with arthritis. The potential anti-inflammatory effect of acupotomy should be considered, as the degrees of joint swelling and pain are important for assessing the response to treatment in arthritis.^[41]^

**Figure 5:**
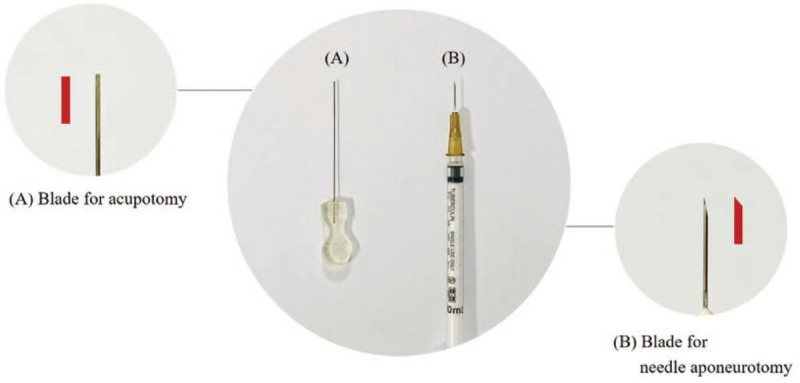
Shape of needle blades for acupotomy (A) and needle aponeurotomy (B).

Acupotomy is similar to needle aponeurotomy (NA), which has been performed for the treatment of Dupuytren's contracture.^[42]^ It is performed to incise the adhered tissue, and 25-gauge, 16-mm long sharp injection needles are usually used. However, it is known that NA is not effective for the treatment of contracture in the absence of Dupuytren's disease or for capsular contractures of the PIP joint. In addition, approximately 20% of patients showed adverse tissue damage such as skin fissure or transient dysesthesia.^[42–44]^ Conversely, as the acupotomy needle has a thick, flat-head and cylindrical body, acupotomy is safer in terms of tissue damage than NA and is used to treat a wider range of skeletomuscular diseases (Fig. 5).^[11]^

There are a few limitations in this study, including a small sample, measurement without a functional rating scale, and the possibility of natural healing over time. However, to our knowledge, this was the first report of post-immobilization contracture needling treatment. We speculate that there is a causal association between the performed acupotomy and contracture improvement, as the symptoms did not improve with conservative treatment and were only relieved after the acupotomy. Both patients were satisfied with the improvement in finger motion and pain. Notwithstanding its limitations, this study does suggest that carefully used acupotomy can be an efficient treatment for post-immobilization joint contracture.

## Author contributions

**Conceptualization:** Sang-Hoon Yoon, Kyongha Cho.

**Funding acquisition:** Sungha Kim.

**Investigation:** Sang-Hoon Yoon, Eunji Lee, Byeongjo Kwon.

**Project administration:** Jiyun Cha, Sungha Kim.

**Supervision:** Jiyun Cha, Sungha Kim.

**Writing – original draft:** Sang-Hoon Yoon, Jiyun Cha.

**Writing – review & editing:** Jiyun Cha, Sungha Kim.

## Supplementary Material

Supplemental Digital Content
