# Supplementary material for: Acupotomy treatment for finger joint contracture after immobilization: Case report
Source: Medicine (Baltimore). 2021 Mar 12;100(10):e24988. doi: 10.1097/MD.0000000000024988 (PMC7969265; doi:10.1097/MD.0000000000024988)
Supplement: Supplemental Digital Content [file medi-100-e24988-s001.docx]

**Checklist for items in STRICTA 2010**

| **Item** | **Detail** |
| --- | --- |
| **1. Acupuncture rationale** | 1a) Style of acupuncture: The style of acupotomy treatment used in this study was based on a recent study as shown in 1b. |
|  | 1b) Reasoning for treatment provided, based on historical context, literature sources, and/or consensus methods, with references where appropriate: The authors performed acupotomy treatment with reference to Ji-Cheol Jeong et al.'s previous treatment of Hand Osteoarthritis (Ji-Cheol Jeong, Sang-Hoon Yoon, Shin-Ae Kim. Effect of Miniscalpel-Acupuncture for Hand Osteoarthritis: Case Report. Journal of Korean Medicine Rehabilitation 2018;28(4):125-30.) and Liu F et al.'s report that acupotomy treatment release adhesions or contractured tissue( Liu F, Zhou F, Zhao M, Fang T, Chen M, Yan X. Acupotomy Therapy for Chronic Nonspecific Neck Pain: A Systematic Review and Meta-Analysis. Evid-Based Complement Altern Med ECAM. 2017;2017:6197308). |
|  | 1c) Extent to which treatment was varied: Each patient received individualized acupotomy treatments focused on symptoms. |
| **2. Details of needling** | 2a) Number of needle insertions per subject per session (mean and range where relevant): 1–2. |
|  | 2b) Names (or location if no standard name) of points used (uni/bilateral): Case 1: the needles were inserted in the first metacarpophalangeal joint and interphalangeal joint where tenderness appeared (left hand). Case2: the needles were inserted in the second finger proximal interphalangeal joint where tenderness appeared (right hand). |
|  | 2c) Depth of insertion, based on a specified unit of measurement, or on a particular tissue level: Within 0.5 cm, adhesions in the articular capsule. |
|  | 2d) Response sought (e.g. *de qi* or muscle twitch response): None. |
|  | 2e) Needle stimulation (e.g. manual, electrical): Manual. |
|  | 2f) Needle retention time: Post-procedural removal. |
|  | 2g) Needle type (diameter, length, and manufacturer or material): An acupotomy needle (0.5 mm diameter, 50 mm long; Dongbang Dochim, Dongbang acupuncture needle company, Korea). |
| **3. Treatment regimen** | 3a) Number of treatment sessions: Case 1: four sessions for 3 months. Case 2: six sessions for 3 months. |
|  | 3b) Frequency and duration of treatment sessions: Average, one session per 2–3 weeks. |
| **4. Other components of treatment** | 4a) Details of other interventions administered to the acupuncture group (e.g. moxibustion, cupping, herbs, exercises, lifestyle advice): Case 1: manual therapy. Case 2: acupuncture. |
|  | 4b) Setting and context of treatment, including instructions to practitioners, and information and explanations to patients: Before each treatment, the doctor of Korean medicine measured the range of motion of the joint. Patients were informed that pain may occur after treatment. |
| **5. Practitioner background** | 5) Description of participating acupuncturists (qualification or professional affiliation, years in acupuncture practice, other relevant experience) : Case 1: After graduating from a 6-year college, the practitioner received a Korean Medicine Doctor license and has 6 years of experience in acupotomy practice. Case 2: After graduating from a 6-year college, the practitioner received a Korean Medicine Doctor license and has 8 years of experience in acupotomy practice. |
| **6. Control or comparator interventions** | 6a) Rationale for the control or comparator in the context of the research question, with sources that justify this choice: Not applicable because this is a case report. |
|  | 6b) Precise description of the control or comparator. If sham acupuncture or any other type of acupuncture-like control is used, provide details as for Items 1 to 3 above: Not applicable because this is a case report. |

Note: This checklist, which should be read in conjunction with the explanations of the STRICTA items, is designed to replace [CONSORT 2010’s item 5](http://www.consort-statement.org/consort-statement/3-12---methods/item5_interventions/) when reporting an acupuncture trial.
